# Prescriptive analytics applied to brace treatment for AIS: a pilot demonstration

**DOI:** 10.1186/1748-7161-10-S1-O64

**Published:** 2015-01-19

**Authors:** Eric Chalmers, Doug Hill, Vicky Zhao, Edmond Lou

**Affiliations:** 1 University of Alberta, Edmonton, AB T6G 2R3, Canada; 2 Alberta Health Services, Canada

## Objectives

Prescriptive analytics is a concept which combines statistical and computer science underpinnings to prescribe an optimal course of action, based on predictions of possible future events. This concept was used to recommend optimal in-brace correction for scoliosis patients. Our objectives were to estimate the efficacy of these recommendations, and formulate improved brace design protocols.

## Methods

A fuzzy model (Chalmers et al, 2013) was developed using data from 90 AIS patients who had finished treatment (60 full-time braces and 30 nighttime. Rates of 6-degree-or-more progression were 53% for daytime braces and 30% for nighttime). The model used clinical measurements taken at the start of treatment to predict whether a given patient's deformity will progress during treatment.

The model predicted individual patients' outcomes for a range of in-brace corrections. These predictions were used to recommend the patient's 'optimal' in-brace correction - the point of diminishing returns, where increasing correction no longer improved the predicted outcome.

The efficacy of the recommendations was estimated using a technique called 'clinical trial simulation' (Chi et al, 2012). This technique uses a statistical model to predict progression rate under the model-recommended treatment, and compares it to the progression rate, observed retrospectively, under the actual treatment.

## Results

Model-recommended corrections ranged from 20%-58% for daytime braces and 65%-130% for nighttime braces, roughly corresponding with previous literature. Interestingly, in 37% of cases the recommendation was less than the correction which had actually been applied, suggesting some opportunity for less aggressive (more comfortable) braces without compromising treatment outcome.

The clinical trial simulation estimated 26% fewer progressive cases using the model-recommended in-brace correction, over the actual correction observed retrospectively in the charts. The patients whose correction decreased under the model's recommendation did not show an increased progression rate.

## Conclusions

Optimal correction may be less than the maximum achievable correction. The preliminary results suggest that considering model-generated recommendations during brace fitting could improve outcomes. Future work will expand the system to recommend wear-times as well as corrections, improving its clinical relevance. We hope this pilot demonstration will promote development of model-based decision support in scoliosis treatment, and prompt discussion on its efficacy and future role.

